# *Drosophila melanogaster* as a Model Organism for Obesity and Type-2 Diabetes Mellitus by Applying High-Sugar and High-Fat Diets

**DOI:** 10.3390/biom12020307

**Published:** 2022-02-14

**Authors:** Nieves Baenas, Anika E. Wagner

**Affiliations:** 1 Institute of Nutritional Medicine, University of Lübeck, Ratzeburger Allee 160, 23538 Lubeck, Germany; nieves.baenas@um.es; 2 Institute of Nutritional Sciences, Justus-Liebig University, Wilhelmstrasse 20, 35392 Giessen, Germany

**Keywords:** fruit fly, metabolic dysfunction, obesity diets, obesity-related diseases

## Abstract

Several studies have been published introducing *Drosophila melanogaster* as a research model to investigate the effects of high-calorie diets on metabolic dysfunctions. However, differences between the use of high-sugar diets (HSD) and high-fat diets (HFD) to affect fly physiology, as well as the influence on sex and age, have been seldom described. Thus, the aim of the present work was to investigate and compare the effects of HSD (30% sucrose) and HFD (15% coconut oil) on symptoms of metabolic dysfunction related to obesity and type-2 diabetes mellitus, including weight gain, survival, climbing ability, glucose and triglycerides accumulation and expression levels of *Drosophila* insulin-like peptides (dIlps). Female and male flies were subjected to HSD and HFD for 10, 20 and 30 days. The obtained results showed clear differences in the effects of both diets on survival, glucose and triglyceride accumulation and dIlps expression, being gender and age determinant. The present study also suggested that weight gain does not seem to be an appropriate parameter to define fly obesity, since other characteristics appear to be more meaningful in the development of obesity phenotypes. Taken together, the results demonstrate a key role for both diets, HSD and HFD, to induce an obese fly phenotype with associated diseases. However, further studies are needed to elucidate the underlying molecular mechanisms how both diets differently affect fly metabolism.

## 1. Introduction

Overweight and obesity are part of an increasing global epidemic that affects high- and, increasingly, also low- and middle-income countries. In 2016, the World Health Organization (WHO) has estimated that more than 1.9 billion adults have been overweight with 650 million out of these being obese [1]. Overweight and obesity are linked to the development of various non-communicable diseases (NCDs) including Diabetes mellitus type 2 (T2DM), cardiovascular diseases, metabolic syndrome and cancer [2], resulting in an increased burden and the possibility of a collapse of global healthcare systems. Therefore, it is of high importance to develop new, effective and implementable pharmacological and non-pharmacological therapies.

The global rise in the incidence of obesity and obesity-related disorders has triggered the need for simple research models to elucidate the underlying mechanisms or dysfunctions implicated in metabolic diseases. The fruit fly *Drosophila melanogaster* has been successfully used for several decades as a model organism mainly in genetic research due to the fact that it conserves approximately 75% of all known human disease-related genes [3] and possesses tissues, organs and systems analogous to those involved in human obesity and associated metabolic complications [4]. Thus, the fruit fly resembles an ideal research model to investigate the pathophysiological mechanisms with regard to obesity-related disorders. It has been demonstrated that the fruit fly exhibits similar metabolic functions as mammals, including the maintenance of glucose homeostasis, lipid storage and mobilization and the regulation of food intake. In *D. melanogaster*, nutrients are digested and absorbed in the midgut and then stored in the fat body and peripheral oenocytes, representing mammalian hepatic and adipose tissues [5]; thus, overnutrition is also associated with increased fat storage in flies. Besides, *Drosophila* insulin-like peptides (dIlps), homologous to mammalian insulin, are the main factors involved in metabolic homeostasis, insulin resistance and hyperglycemia (increased hemolymph glucose concentrations) [6].

In addition, the use of the fruit fly as a model organism in obesity and/or metabolic dysfunctions combines several advantages, including relatively cost-effective maintenance, high production of progenies within a short period of time, the presence of tissues and organs as well as the presence of various disease-associated genes and the possibility to mimic diet-induced disorders. High-sugar diets (HSD), as well as high-fat diets (HFD), have been applied in *D. melanogaster* to induce either an obese phenotype and/or metabolic dysfunction, being reflected in T2DM-like symptoms such as insulin resistance and high levels of sugar (glucose/trehalose) [6,7,8]. Data comparing both diet types with regard to the modulation of metabolic parameters in fruit flies are limited. In the present study, we therefore tested whether HSD and HFD feeding result in different outcomes of obesity and/or metabolic dysfunction-related parameters in the fruit fly. The obtained results may provide further information on the specific use of either diet in the context of answering specific research questions in connection with obesity and/or metabolic dysfunction.

## 2. Materials and Methods

### 2.1. Fly Strains and Husbandry

The wild-type strain w^1118^ (Bloomington Drosophila Stock Center, Bloomington, IN, USA; #5905) was used in all experiments. Flies were maintained in a humidified (60%) and temperature-controlled (25 °C) climate chamber (Memmert, HPP400, Buechenbach, Germany), with a 12 h/12 h light–dark cycle on standard fly medium (CT) according to Wagner et al. [9]. Age-matched flies were obtained from synchronized eggs as described by Linford and co-workers [10] with slight modifications. Newly hatched flies were allowed to mate for two days and were then separated according to their sex. Male and female flies were transferred into vials containing the corresponding experimental diets. In the present study, flies were kept on a control, high-sugar (HSD) or high-fat diet (HFD). The compositions of the diets are listed in Table 1.

### 2.2. Experimental Design

Male and female flies were exposed to the different experimental diets listed in Table 1 for 10, 20 and 30 days. Thirty flies were sorted into one vial and the flies were transferred to vials containing fresh medium every two to three days. All experiments were repeated with three biological replicates and performed in compliance with the authors’ institute’s policy on animal use and ethics.

### 2.3. Survival

Survivorship of w^1118^ flies was documented over the study period of 30 days. During the flipping procedure every two to three days, the numbers of dead flies per vial were recorded.

### 2.4. Climbing

At the end of the different treatment periods, the climbing ability of flies exposed to the different experimental diets was assessed according to Gargano and colleagues [11]. Briefly, ten flies per vial and treatment were transferred into an empty vial, the vial was tapped to a surface three times to bring all flies to the bottom and a picture was taken after three seconds. This procedure was repeated 10 times, while flies were allowed to recover between the taps for 30 s. The relative climbing index was calculated by dividing the climbing height into five equal sections and scoring them from zero (at the bottom) to four (highest section). The height climbed was calculated by summing up the number of flies per treatment in the different sections.

### 2.5. Fly Weights

On days 10, 20 and 30, flies were transferred into a pre-weighed empty vial and the mean weight of a single fly was calculated by dividing the total fly weights by the number of flies per vial.

### 2.6. Glucose and Triglyceride Levels

Flies fed on different experimental diets were analyzed for their content of glucose and triglycerides. Five flies per vial and treatment were transferred into a 2 mL Eppendorf tube and homogenized in 250 μL PBS containing Triton-X-100 (1% *v*/*v*) in a Tissue Lyser at a frequency of 1/s = 25 for 2 × 3 min (Qiagen, Hilden, Germany). Then, the fly lysates were centrifuged (Heraeus Multifuge X3 FR; Thermo Fisher Scientific Life Technologies GmbH, Carlsbad, CA, USA) at 5000× *g*, 4 °C for 10 min. The supernatant was transferred into fresh 0.5 mL tubes and either used immediately or stored at −80 °C until further use. To prevent the interference of proteins in the glucose assay, samples were heated at 70 °C for 5 min, immediately cooled down and centrifuged at 16,000× *g*, 4 °C for 10 min. Supernatants were used immediately for analysis or stored at −80 °C until further use. The samples were diluted in homogenization buffer and subjected to glucose and triglyceride analysis. Glucose levels were detected by Fluitest GLU (Analyticon Biotechnologies AG, Lichtenfels, Germany) calculated via a glucose standard curve (0–100 mg/dL) prepared from a glucose standard (100 mg/dL). In brief, samples and glucose standards were mixed with the reaction buffer, incubated at 37 °C for 15 min and measured for absorbance (540 nm) in a plate reader (SpectraMax^®^iD3, Molecular Devices, San José, CA, USA). Triglyceride levels were detected by Fluitest TG (Analyticon Biotechnologies AG, Lichtenfels, Germany) calculated via a glycerol standard curve (0–200 mg/dL) prepared from a glycerol standard (equivalent to a concentration of 200 mg/dL triglycerides). In brief, samples and glycerol standards were mixed with the reaction buffer, incubated at 37 °C for 5 min and measured for absorbance (540 nm) in a plate reader (SpectraMax^®^iD3, Molecular Devices, San José, CA, USA). Triglyceride and glucose levels were normalized to fly weights. The analyses were performed in duplicate for each replicate per treatment.

### 2.7. Real-Time PCR Analysis

Total RNA from whole flies exposed to the experimental diet for 30 days was extracted using the TriFast reagent (Peqlab Biotechnologie, Erlangen, Germany) and subjected to DNAse digestion (AMPD1 Kit, Sigma-Aldrich, Taufkirchen, Germany) according to the manufacturer’s instructions. Prior to RNA isolation, 5 flies per sample were homogenized in a Tissue Lyser at a frequency of 1/s = 25 for 2 × 3 min, and RNA concentrations and purity were determined in a NanoDrop (NanoDrop 2000, ThermoScientific, Waltham, MA, USA). Gene expression levels were analyzed by two-step real-time PCR. cDNA synthesis was performed using an OligodT primer (Promega, Mannheim, Germany), RNase inhibitor Ribolock (Promega), dNTPs (Fisher Scientific, Schwerte, Germany) and revert Aid H Minus Reverse Transcriptase plus reaction buffer (Thermo Scientific) in a thermocycler (Biometra, Göttingen, Germany). For real-time PCR, a PerfeCTa© SYBR© Green Super Mix (Quantabio, Beverly, MA, USA) was applied, and samples were detected in a StepOnePlus System (Applied Biosystems, Waltham, MA, USA) using the primers listed in Table 2. Relative mRNA quantification was performed by applying the ∆∆Ct method. rpl32 served as the housekeeping gene.

### 2.8. Statistical Analysis

Statistical analysis was performed using Prism 8 (Version 8.4.3; GraphPad Software, LLC, San Diego, CA, USA). The data were tested for normality of distribution (Kolmogorov–Smirnov or Shapiro–Wilk) and subjected to an unpaired *t*-test (normal distribution) and a Mann–Whitney-U test (non-parametric test), respectively. Data are presented as means ± SEM. Fly survival was calculated using the Kaplan–Meier approach and analyzed for significant differences by applying the log-rank test. The correlation coefficient (r) was calculated by the Pearson correlation. Multiple comparisons of all variables were performed by mixed-effects analysis. Significance for all analyzed data was accepted at *p* < 0.05.

## 3. Results

### 3.1. Survival

**Figure 1:**
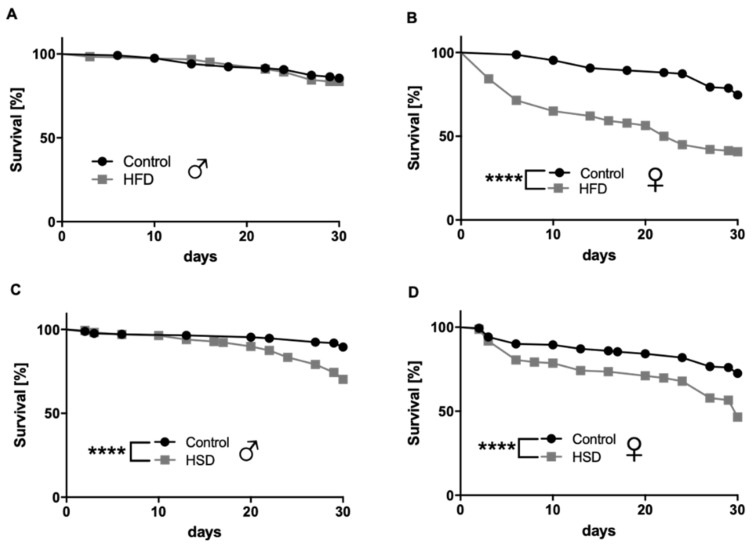
Survival rates of w^1118^ *Drosophila melanogaster* on different diets. Male flies being reared on a high-fat diet (HFD) for 30 days did not show differences in survival rates compared to male flies on a control diet (**A**). Female flies reared on a HFD for 30 days exhibited a significant decrease in their survival rates compared to the corresponding controls (**B**). The survival rates of male (**C**) and female (**D**) flies significantly decreased by feeding a high-sugar diet (HSD) in comparison to flies fed a control diet. Data represent the mean from three biological replicates (*n* = 75). **** indicates significant differences (*p* < 0.0001) calculated by the log-rank test.

Female flies fed an HFD containing 15% coconut fat, compared to 0% coconut fat in the control diet, exhibited a significant decrease in survival up to experimental day 30 (*p* < 0.0001; Figure 1B) while this was not true for male flies (*p* = 0.6194; Figure 1A). Both male and female w^1118^ flies on an HSD with 30% sugar showed a significant decrease in survival up to experimental day 30 compared to flies on a control diet with 10% sugar (*p* < 0.0001; Figure 1C,D).

### 3.2. Weight Gain

**Figure 2:**
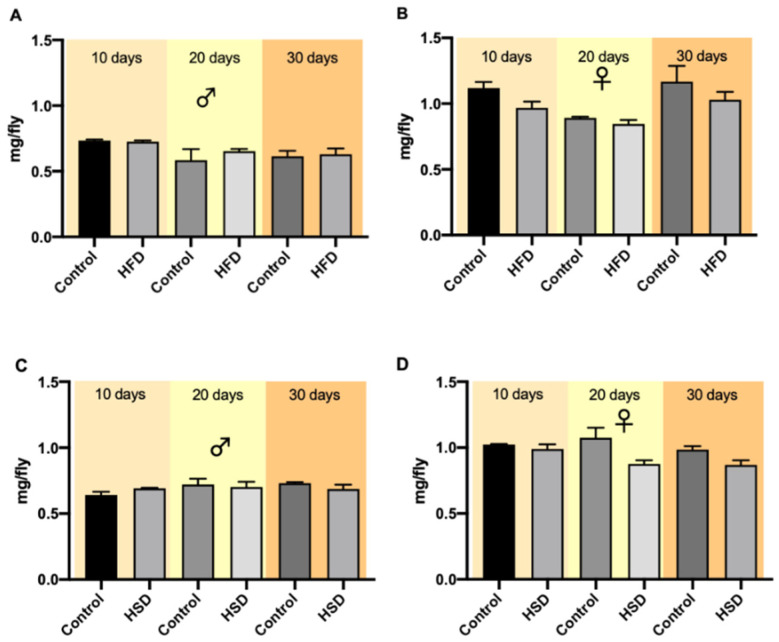
The weights of w^1118^ *Drosophila melanogaster* exposed to different diets and time periods. Bars show the mean weights of male (**A**) and female (**B**) flies reared on a high-fat diet (HFD) and male (**C**) and female (**D**) flies on a high-sugar diet (HSD) on days 10, 20 and 30, respectively. Data are presented as mean ± SEM from three biological replicates (*n* = 75).

Flies were weighed after treatment on days 10, 20 and 30. The weights of both male and female flies on a control diet and either on HFD or HSD did not differ significantly at the different measuring points (Figure 2A–D). A comparison of all variables (sex, age, time) revealed no significant differences (Figures S1 and S2, Tables S1 and S2).

### 3.3. Climbing

**Figure 3:**
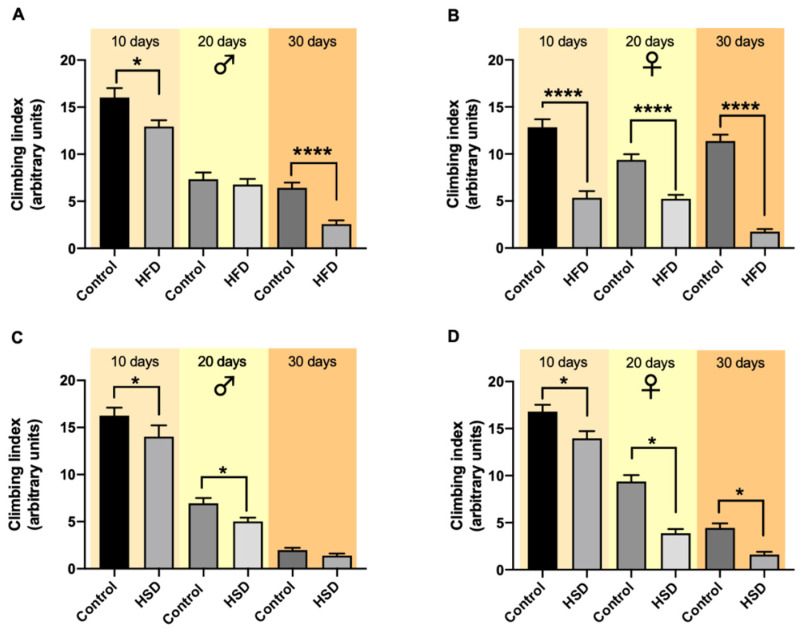
The climbing ability of w^1118^ *Drosophila melanogaster* being reared on different diets and time periods. The exposure of male flies to a high-fat diet (HFD) for 10 and 30 days significantly decreased the flies’ climbing ability compared to the flies on a control diet, while after 20 days on HFD no significant changes were detected (**A**). In female flies, 10-, 20- and 30-day treatment with HSD resulted in a significant decrease in the climbing index compared to the corresponding control flies (**B**). Male flies on a high-sugar diet (HSD) exhibited a significant decrease in their climbing ability compared to flies on a control diet after 10 and 20 days while this difference was not present after HSD exposure for 30 days (**C**). Feeding female flies HSD for 10, 20 and 30 days resulted in a significant decrease in their climbing index compared to flies on a control diet (**D**). Bars present the mean ± SEM from three biological replicates (*n* = 30). * (*p* < 0.05) and **** (*p* < 0.0001) indicates significant differences compared to the corresponding control flies calculated by an unpaired *t*-test or Mann–Whitney test (non-parametric).

Male and female w^1118^ flies were tested for their climbing ability after 10, 20 and 30 days reared either on HSD or HFD. In male flies, HFD caused a significant decrease in the climbing index after both 10 and 30 days, while after 20 days, the climbing index did not differ between flies on a control diet and HFD (Figure 3A). A comparison of all variables (sex, age, time) revealed no significant differences (Figures S3 and S4, Tables S3 and S4).

In female flies exposed to HFD for 10, 20 and 30 days, the flies’ climbing ability significantly decreased compared to the corresponding control flies (Figure 3B). Moreover, in flies exposed to HSD, the climbing ability was significantly lower after 10 and 20 days in male flies (Figure 3C) and after 10, 20 and 30 days in female flies (Figure 3D) in comparison to flies on a control diet.

### 3.4. Glucose and Triglycerides

**Figure 4:**
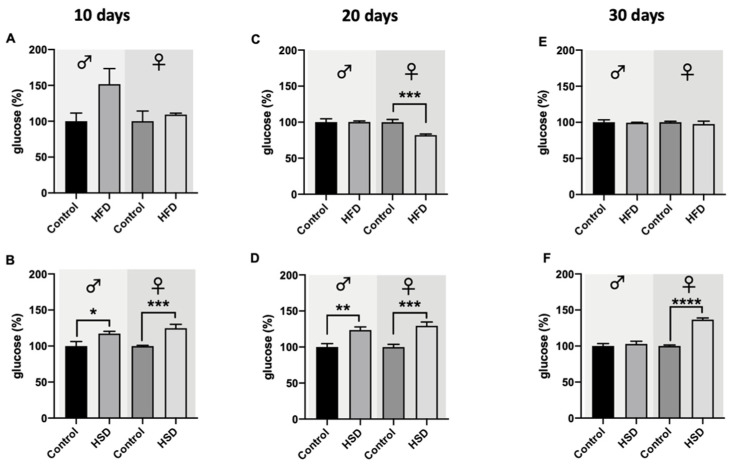
Glucose levels of whole-fly extracts from w^1118^ *Drosophila melanogaster*. In both male and female flies reared on a high-fat diet (HFD) for 10 days (**A**), the glucose levels did not differ compared to the flies on a control diet. Male and female flies fed a high-sugar diet (HSD) for 10 days (**B**) exhibited a significant upregulation of the glucose levels compared to the corresponding control flies. After 20 days on HFD, the glucose levels in male flies remained at a similar level as the control flies, while female flies on HFD exhibited significantly lower glucose levels in comparison to control flies (**C**). Male and female *Drosophila melanogaster* reared on HSD for 20 days significantly increased their glucose levels compared to the corresponding control flies (**D**). After 30 days on HFD, no changes in comparison to control flies regarding glucose levels in male and female flies were detected (**E**). In male flies, treatment with HSD for 30 days did not affect the flies’ glucose levels, while in female flies, a significant increase in glucose levels compared to control animals was observed (**F**). Bars represent the mean ± SEM from three biological replicates (*n* = 3 × 5 flies). * (*p* < 0.05), ** (*p* < 0.01), *** (*p* < 0.001) and **** (*p* < 0.0001) indicates significant differences compared to the corresponding control flies calculated by an unpaired *t*-test.

In flies exposed to HFD for 10 days, the glucose levels were not affected in both sexes (Figure 4A). However, in male flies reared on HFD, a trend towards upregulation of glucose levels with borderline significance (*p* = 0.0509) was observed. In the case where flies were exposed to HSD for 10 days, the glucose levels significantly increased in both male and female flies in comparison to the corresponding control flies (Figure 4B). After 20 days on HFD, male flies did not exhibit a change in their glucose levels compared to control-diet-fed flies, while female flies on HFD for 20 days showed significantly lower glucose levels compared to the control flies (Figure 4C). A 20-day duration of exposure to HSD resulted in a significant increase in glucose levels in both male and female flies. In male and female flies reared on HFD for 30 days, no significant changes in glucose levels in comparison to flies on a control diet were detected (Figure 4E). In the case of flies reared for 30 days on HSD, male flies did not differ in their glucose levels compared to the corresponding controls, while female flies on HSD exhibited a significant increase in glucose compared to female flies on a control diet (Figure 4F).

**Figure 5:**
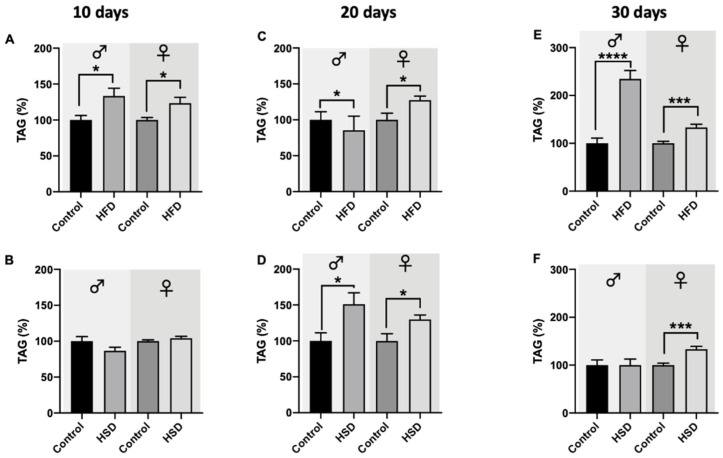
Triglyceride (TAG) levels of whole-fly extracts from w^1118^ *Drosophila melanogaster*. In both male and female flies reared on a high-fat diet (HFD) for 10 days (**A**), TAG levels were significantly increased compared to the corresponding control flies. Male and female flies fed a high-sugar diet (HSD) for 10 days did not show any changes compared to flies on a control diet (**B**). After 20 days on HFD (**C**) and HSD (**D**), TAG levels in male and female flies significantly increased compared to control animals. TAG levels significantly increased compared to flies on a control diet in both male and female *Drosophila melanogaster* on HFD for 30 days (**E**). In male flies, treatment with HSD for 30 days did not affect the flies’ TAG levels, while in female flies, a significant increase in TAG levels compared to control animals was present (**F**). Bars represent the mean ± SEM from three biological replicates (*n* = 3 × 5 flies). * (*p* < 0.05), *** (*p* < 0.001) and **** (*p* < 0.0001) indicate significant differences compared to the corresponding control flies calculated by an unpaired *t*-test and a Mann–Whitney test (non-parametric), respectively.

Male and female w^1118^ flies reared on HFD for 10 days showed significant induction of their triglyceride levels compared to the corresponding control flies (Figure 5A). Exposure to HSD for 10 days, however, did not result in any significant changes regarding the triglycerides levels in both male and female *D. melanogaster*, in comparison to flies on a control diet (Figure 5B). After 20 days on HFD, male flies exhibited significantly lower triglyceride levels compared to their control counterparts while female flies significantly increased their triglyceride levels (Figure 5C). Interestingly, male and female flies reared on HSD for 20 days showed significant upregulation of their triglyceride levels compared to flies on a control diet (Figure 5D). Curiously, male flies on HSD increased their triglyceride levels by 50% which was, however, not present under HFD exposure. Female flies, however, exhibited a similar triglyceride level increase (+~25%) by both diets, HSD and HFD, compared to flies on a control diet. The triglyceride levels of male flies exposed to HFD for 30 days significantly increased by more than 100%, while in female flies, HFD significantly increased the triglyceride levels by ~25% compared to the controls (Figure 5E). This increase is similar to the extent observed after 10 and 20 days of HFD feeding of female flies. No changes in triglyceride levels were detected in male flies on HFD and control diets, while in female flies, a significant increase in triglyceride levels was detected (Figure 5F).

### 3.5. Drosophila Insulin-like Peptides

**Figure 6:**
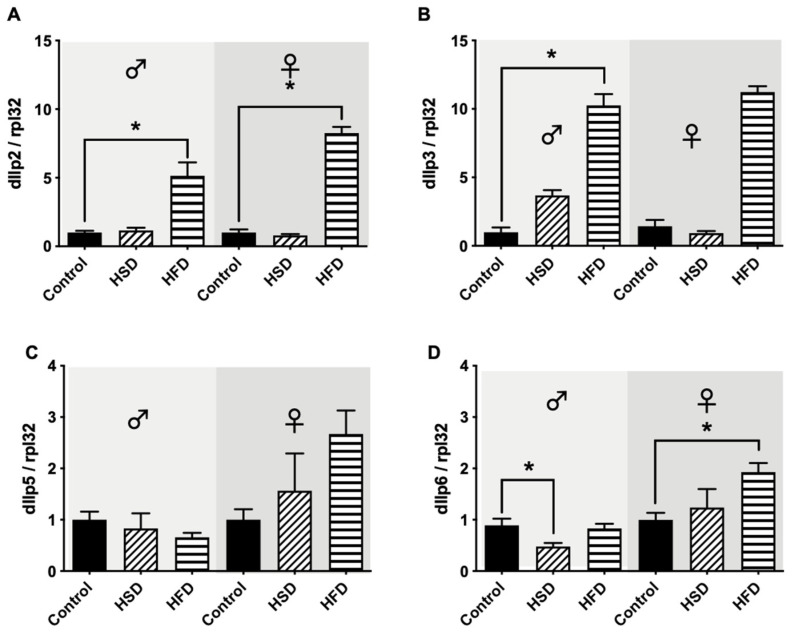
Relative mRNA expression levels of *Drosophila* insulin-like peptides (DILPs) in w^1118^ flies reared on either a control diet, high-fat diet (HFD) or high-sugar diet (HSD) for 30 days. HFD significantly increased the relative mRNA levels of dIlp2 in male and female flies, while HSD feeding did not affect dIlp2 levels compared to the corresponding controls (**A**). In male flies, HFD but not HSD significantly increased dIlp3 levels in comparison to flies on a control diet, while in female flies, neither HSD nor HFD caused significant changes in comparison to the controls (**B**). The relative expression levels of dIlp5 were not affected by either HSD or HFD in both male and female flies (**C**). With regard to the relative mRNA levels of dIlp6, HSD significantly decreased dIlp6 in male flies, while in female flies, HFD significantly increased the dIlp6 levels in comparison to control animals (**D**). Bars represent the mean ± SEM from three biological replicates (*n* = 3 × 7 flies). * (*p* < 0.05) indicates significant differences compared to the corresponding control flies calculated by ANOVA or Kruskal-Wallis test, followed either by Dunnett’s or Bonferroni’s post-hoc test, or Dunn’s test, respectively.

After 30 days on either HSD or HFD, male and female flies showed a significant increase in the mRNA expression levels of dIlp2 in comparison to flies reared on a control diet (Figure 6A). Similar results were found in male *D. melanogaster* regarding the mRNA expression levels of dIlp3. Following HSD exposure, dIlp3 mRNA levels remained unchanged compared to flies on a control diet, while HFD exposure significantly upregulated the expression levels of dIlp3. In female flies, however, no significant changes in dIlp3 expression levels following HSD and HFD exposure could be detected (Figure 6B). dIlp5 expression levels were not affected by HSD and HFD compared to flies on a control diet in both male and female flies (Figure 6C). Regarding the mRNA expression levels of dIlp6, HSD exposure for 30 days resulted in significant downregulation compared to controls in male *D. melanogaster*, while HFD did not change dIlp6 expression. In female flies, however, HSD did not affect dIlp6 mRNA levels, while HFD for 30 days resulted in a significant increase in dIlp6 expression in comparison to the corresponding control flies (Figure 6D).

## 4. Discussion

In the present work, the effects of the most common diets used to elicit obesity, a high-sugar diet (HSD) containing 30% sucrose and a high-fat diet (HFD) containing 15% coconut oil [12], have been evaluated for parameters of metabolic dysfunction in connection with the potential risk of developing obesity and T2DM in male and female flies reared on these diets for 10, 20 and 30 days.

Regarding obesity and aging in *D. melanogaster,* controversial results have been published. Some authors reported a significant reduction of the lifespan in flies treated with HSD [13,14] or HFD [15,16], and others revealed a remarkable extension after HSD [17]. In the present study, both dietary modifications produced decreases in lifespan, while the effect was more pronounced in females than in males, with the latter only being affected by HSD from 20 days of treatment. Sex differences in response to nutritional variations have been recently and widely reported in *D. melanogaster* [18,19,20,21], as well as in a range of species; however, the genetic and molecular bases for this dimorphism are often unknown. Previous investigations point to (a) differences in reproductive gene responses mediated by the classical nutrient-sensing pathway IIS/TOR [22] and (b) sexual dimorphism in the intestinal tract [23] as the main factors influencing the adaptive responses of *D. melanogaster* to the dietary environment. Accordingly, recent studies revealed mated females as more sensitive than males to HSD and HFD, suggesting higher intestinal stem cell proliferation in mated females as a responsible factor in reducing the lifespan and increasing nutrient absorption [18,19,23,24,25]. Undoubtedly, adaptation to reproductive functions is the main cause of sex differences, as carbohydrates are the source of energy that males need for mating, while females mainly invest in proteins for providing eggs [21,26]. Previous works have also shown that females especially were more sensitive to HFD and HSD compared to males [19,25]. Moderate decreases in the fly lifespan have been related to different physiological processes affected by HSD and HFD diets, such as fat accumulation and hyperglycemia, damage to nephrocytes, decreases in immunity, heart dysfunction and gut homeostasis disruption, among others [27].

In humans, excess weight gain is an important parameter in the development of obesity. Nevertheless, this fact has scarcely been studied in *D. melanogaster*. Only a few publications have demonstrated an increase in weight in flies exposed to obesity diets, such as HFD [8], while others reported a decrease with HFD [15] and HSD [14]. Besides, certain review articles regarding obesity in *Drosophila* did not report enlightening information on this parameter [12,27,28]. In the present work, fly weights were not affected by the different diets. According to the literature, several factors influence changes in the body weight of *D. melanogaster* fed obesity diets, including age [14], mating [15], insulin/IGF signaling pathway modulation [29] and leptin expression in the brain [30]. In our work, the sum of those diet effects may explain why fly weights did not change over the 30 days of the experiment. For instance, mating may have influenced the weight in females, as it has been described that diets supplemented with fat and/or sugar affect oogenesis, a decrease in the size of the ovaries and, consequently, weight [18]. However, the increased lipid and/or carbohydrate accumulation caused by increased insulin signaling would counteract this effect [29]. Besides, decreased growth in male and female flies caused by obesity diets could generally explain the lack of weight gain [31].

The evaluation of climbing ability has been widely used to determine the effects of dietary treatments on the physiology, fitness and behavior of flies. According to our results, this parameter progressively diminished in flies during normal aging. However, it has also been shown to decline with exposure to obesity diets [8,15,28]. In our flies, a clear negative effect of both HSD and HFD on climbing ability was observed, while female flies were significantly more affected than males. These behavioral differences may be due to the described sex-specific differences in the octopaminergic neuron activity, one of the main factors responsible for generating different endurance responses in flies [32].

As in humans, flies’ main circulating energy sources are sugars, with trehalose (the predominant one) and glucose found in the hemolymph [33]. The disaccharide trehalose is accumulated in the fly body without negative effects, being converted into glucose when there are energy needs [27]. Both trehalose and glucose have been reported to increase with HSD exposure in *D. melanogaster* [7]. In our study, only flies exposed to HSD showed a significant increase in glucose levels, reflecting a type of hyperglycemia, a defining sign of T2DM. Higher glucose levels were more pronounced in females than in males, where this increase was not found after a 30-day treatment with HSD. This may be explained by sex-dependent differences in the gut that slowed the intestinal transit and enhanced the nutrient absorption in mated females compared to males [24,34]. Even though some authors reported hyperglycemia after HFD consumption [8,16], HFD did not result in an accumulation of glucose in our flies. Nevertheless, all flies treated with HFD exhibited increased TAG levels that further increased with age. DiAngelo and Birnbaum reported that TAG accumulation was promoted by insulin signaling in the fat body, the storage organ of the flies. Thus, increased TAG levels following HFD compared to HSD exposure may account for the higher expression levels of dIlps and the consequent regulation of glucose uptake observed in our work, which is supported by a significant positive correlation of higher triglyceride levels with higher glucose levels (Pearson correlation, *r* = 0.7208, *p* = 0.0285) in female flies on HFD. Even though elevated glucose levels have been suggested as fundamental in T2DM development, defects in lipid storage also play an important role in the pathogenesis of this disease [7,8,29].

Fly diets high in carbohydrates (~30%) have been reported to increase the fat content in flies from 50–150%, consequently affecting metabolic homeostasis [27]. After feeding flies with HSD for 20 days, a clear increase in TAG levels was observed (~50%), while significant upregulation after a 30-day-treatment with HSD was only present in females. Thus, HSD affected the glucose homeostasis more than TAG accumulation in the flies, with these results being in line with Nakitto et al. who showed that female flies fed HSD for 10 days had significantly higher glucose levels while TAG levels remained unchanged [35]. Interestingly, male flies exposed to HSD for 30 days did not show an accumulation of either glucose or TAG, nor a reduction in the climbing ability, suggesting that aging in male *D. melanogaster* may result in flies being more resistant to metabolic changes [36]. Sex-dependent responses in TAG accumulation have been described previously, confirming that females develop larger fat cells than males on western diets supplemented with sugar and fat (10–20%) [18], suggesting a key role in successful reproduction [37].

*D. melanogaster* tries to compensate for nutritional abundance with an increased expression of dIlps to increase insulin production, through the activation of the conserved insulin-like growth factors (IGF) signaling pathway [36]. dIlp2, dIlp3 and dIlp5 are expressed predominantly in neuroendocrine cells of adult insects, while dIlp6 is mainly expressed in the fat body. It has been described that specific dIlp genes are independently regulated by dietary interventions and influence different features of metabolism: dIlp2 and 5 are related to glycogen storage, dIlp3 can regulate trehalose storage and dIlp5 and dIlp7 regulate TAG synthesis, suggesting the production of distinct changes in fly metabolism under different obesity diets [38].

In the present study, only flies treated with HFD experienced an increase in dIlps expression. According to DiAngelo and Birnbaum, this fact suggests the activation of insulin signaling in the fat body due to an excess of nutrients, consequently increasing the number of adipocytes and TAG accumulation. It has also been reported that dIlps secretion seems to be regulated by signals from the fat body cells [38]. As flies subjected to HFD did not show increased glucose levels, we suggest enhanced glucose uptake by the fat body in response to elevated insulin secretion. The maintenance of glucose homeostasis in connection with an upregulation of dIlps has also been reported by Alfa and Kim [39].

## 5. Conclusions

The present study explores whether excess sugar and fat consumption through HSD and HFD, respectively, impaired different metabolic functions in flies, finding females to be more susceptible than males. An excess of sugar (HSD) seemed to induce more hyperglycemic effects, while an increased intake of fat (HFD) seemed to affect more insulin signaling pathways. Weight gain appeared to be an inappropriate parameter to define fly obesity, as other, less-studied characteristics, such as deterioration in climbing ability and accumulation of fat or carbohydrates, are more informative for the development of obesity phenotypes. Sex and age are significant factors and need to be considered when establishing fruit fly experiments. Our study supports the evidence of a key role of HSD and HFD in the pathogenesis of obesity and associated diseases. However, further studies are necessary to elucidate the specific underlying molecular mechanisms by which both diet types affect fly metabolism differently.

## Figures and Tables

**Table 1:** Composition of the experimental diets.

Ingredients	Control	High Sugar Diet (HSD)	High Fat Diet (HFD)
Water (mL)	1075	1075	1075
Agar ^1^ (g)	20	20	20
Sucrose ^2^ (g)	100	300	100
Inactive yeast ^3^ (g)	50	50	50
Pure coconut oil ^4^ (g)	0	0	150
Tegosept ^5^ (20% in 95% ethanol) (mL)	15	15	15
Propionic acid ^6^	3	3	3

^1^ Apex via Kisker, Steinfurt, Germany, ^2^ Carl Roth, Karlsruhe, Germany, ^3^ Genesee via Kisker, ^4^ Sigma C1758, Taufkirchen, Germany, ^5^ Apex via Kisker, ^6^ Carl Roth.

**Table 2:** Primer sequences (*Drosophila melanogaster*) used for real-time PCR.

Gene	Forward Primer (5′→3′)	Reverse Primer (5′→3′)	Accession No. (NM_...)
*dIlp2*	AACGAGGTGCTGAGTATGGT	CGAACTCCTGGACAAACTGC	079288
*dIlp3*	ATCCTTATGATCGGCGGTGT	GTTCACGGGGTCCAAAGTTC	140103
*dIlp5*	TGATGGACATGCTGAGGGTT	CATGTGGTGAGATTCGGAGC	206315
*dIlp6*	TGGCGATGTATTTCCCAACAG	CCTTCACTATCCTTTGCAGTACT	130644
*rpl32*	GGCAAGCTTCAAGATGACCA	GTTCGATCCGTAACCGATGT	170461

## Data Availability

The raw data presented in this study are available on request from the corresponding author.

## Supplementary Materials

The following supporting information can be downloaded at: https://www.mdpi.com/article/10.3390/biom12020307/s1, Figure S1: The weights of w^1118^ *Drosophila melanogaster* exposed to different diets and time periods, Figure S2: The weights of w^1118^ *Drosophila melanogaster* exposed to different diets and time periods, Figure S3: The climbing ability of w^1118^ *Drosophila melanogaster* being reared on different diets and time periods, Figure S4: The climbing ability of w^1118^ *Drosophila melanogaster* being reared on different diets and time periods, Table S1: Results for mixed-effects analysis, Table S2: Results for mixed-effects analysis, Table S3: Results for mixed-effects analysis, Table S4: Results for mixed-effects analysis.
