# Supplementary material for: Drosophila melanogaster as a Model Organism for Obesity and Type-2 Diabetes Mellitus by Applying High-Sugar and High-Fat Diets
Source: Biomolecules. 2022 Feb 14;12(2):307. doi: 10.3390/biom12020307 (PMC8869196; doi:10.3390/biom12020307)
Supplement: Supplementary file 1 [file biomolecules-12-00307-s001.zip › biomolecules-1573855-supplementary.pdf]

biomolecules

MDPI

Supplementary Materials

1. Weight
a) High Fat Diet (HFD)

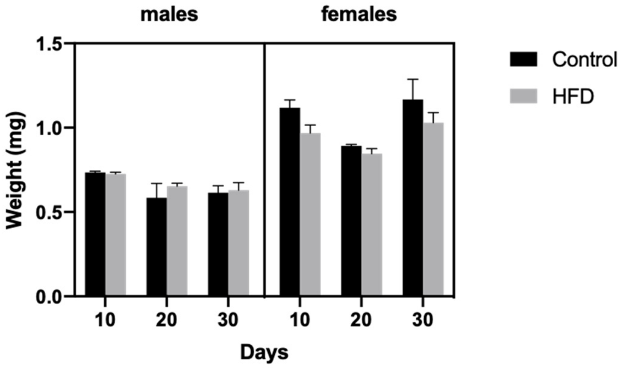

Figure S1. The weights of w[1118] *Drosophila melanogaster* exposed to different diets and time periods. Bars show the mean weights of male and female flies being reared on a control and a high fat diet (HFD) for 10, 20 and 30 days, respectively. Data have been subjected for multiple comparisons to a mixed-effects analysis. Data are presented as mean ± SEM from three biological replicates (n = 75).

Table S1. Results for mixed-effects analysis performed by GraphPad Prism

	Mixed-effects analysis<br>Tabular results				
1	Table Analyzed	Grouped_HFD_Weight			
2					
3	**Mixed-effects model (REML)**	Matching by factor: Diet			
4	Assume sphericity?	Yes			
5	Alpha	0.05			
6					
7	**Fixed effects (type III)**	**P value**	**P value summary**	**Statistically significant (P < 0.05)?**	**F (DFn, DFd)**
8	Time	0.0011	**	Yes	F (2, 33) = 8.504
9	Diet	<0.0001	****	Yes	F (1, 33) = 133.0
10	Gender	0.1598	ns	No	F (1, 33) = 2.069
11	Time x Diet	0.0121	*	Yes	F (2, 33) = 5.057
12	Time x Gender	0.4300	ns	No	F (2, 33) = 0.8660
13	Diet x Gender	0.0299	*	Yes	F (1, 33) = 5.148
14	Time x Diet x Gender	0.9645	ns	No	F (2, 33) = 0.0362
15					
16	**Random effects**	**SD**	**Variance**		
17	Subject	0.000	0.000		
18	Residual	0.09851	0.009705		
19					
20	**Was the matching effective?**				
21	Chi-square, df				
22	P value				
23	P value summary				
24	Is there significant matching (P < 0.05)?	No			
25					
26	**Data summary**				
27	Number of columns	2 x 2			
28	Number of rows (Time)	3			
29	Number of subjects (Subject)	24			
30	Number of missing values	3			

b) High Sugar Diet (HSD)

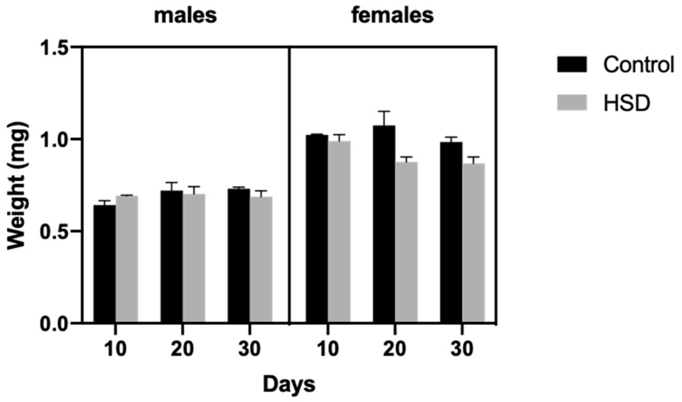

Figure S2. The weights of w[1118] *Drosophila melanogaster* exposed to different diets and time periods. Bars show the mean weights of male and female flies being reared on a control and a high sugar diet (HSD) for 10, 20 and 30 days, respectively. Data have been subjected for multiple comparisons to a mixed-effects analysis. Data are presented as mean ± SEM from three biological replicates ($n = 75$).

Table S2. Results for the mixed-effects analysis performed by GraphPad Prism

	Mixed-effects analysis Tabular results				
1	Table Analyzed	Grouped_HSD_Weight			
2					
3	**Mixed-effects model (REML)**	Matching by factor: Diet			
4	Assume sphericity?	Yes			
5	Alpha	0.05			
6					
7	**Fixed effects (type III)**	**P value**	**P value summary**	**Statistically significant (P < 0.05)?**	**F (DFn, DFd)**
8	Time	0.5699	ns	No	F (2, 24) = 0.5756
9	Diet	<0.0001	****	Yes	F (1, 24) = 180.7
10	Gender	0.0070	**	Yes	F (1, 24) = 8.715
11	Time x Diet	0.0667	ns	No	F (2, 24) = 3.038
12	Time x Gender	0.0685	ns	No	F (2, 24) = 3.004
13	Diet x Gender	0.0111	*	Yes	F (1, 24) = 7.572
14	Time x Diet x Gender	0.5058	ns	No	F (2, 24) = 0.7018
15					
16	**Random effects**	**SD**	**Variance**		
17	Subject	0.000	0.000		
18	Residual	0.06101	0.003723		
19					
20	**Was the matching effective?**				
21	Chi-square, df				
22	P value				
23	P value summary				
24	Is there significant matching (P < 0.05)?	No			
25					
26	**Data summary**				
27	Number of columns	2 x 2			
28	Number of rows (Time)	3			
29	Number of subjects (Subject)	18			
30	Number of missing values	0			

2. Climbing

a) High Fat Diet (HFD)

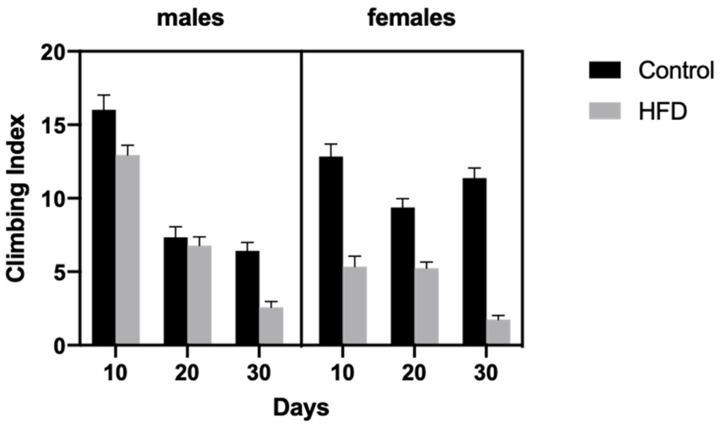

Figure S3. The climbing ability of w^{1118} *Drosophila melanogaster* being reared on different diets and time periods. Bars show the mean climbing index of male and female flies being reared on a control and a high fat diet (HFD) for 10, 20 and 30 days, respectively. Data have been subjected for multiple comparisons to a mixed-effects analysis. Data are presented as mean ± SEM from three biological replicates (*n* = 75).

Table S3. Results for mixed-effects analysis performed by GraphPad Prism:

	Mixed-effects analysis<br>Tabular results				
1	Table Analyzed	Grouped_HFD_Climbing_all			
2					
3	**Mixed-effects model (REML)**	Matching by factor: Gender			
4	Assume sphericity?	Yes			
5	Alpha	0.05			
6					
7	**Fixed effects (type III)**	**P value**	**P value summary**	**Statistically significant (P < 0.05)?**	**F (DFn, DFd)**
8	Time	<0.0001	****	Yes	F (2, 447) = 102.9
9	Gender	0.0063	**	Yes	F (1, 447) = 7.533
10	Control	<0.0001	****	Yes	F (1, 447) = 162.5
11	Time x Gender	<0.0001	****	Yes	F (2, 447) = 36.71
12	Time x Control	<0.0001	****	Yes	F (2, 447) = 12.30
13	Gender x Control	<0.0001	****	Yes	F (1, 447) = 37.13
14	Time x Gender x Control	0.4304	ns	No	F (2, 447) = 0.8447
15					
16	**Random effects**	**SD**	**Variance**		
17	Subject	0.000	0.000		
18	Residual	3.889	15.13		
19					
20	**Was the matching effective?**				
21	Chi-square, df				
22	P value				
23	P value summary				
24	Is there significant matching (P < 0.05)?	No			
25					
26	**Data summary**				
27	Number of columns	2 x 2			
28	Number of rows (Time)	3			
29	Number of subjects (Subject)	232			
30	Number of missing values	5			

b) High Sugar Diet (HSD)

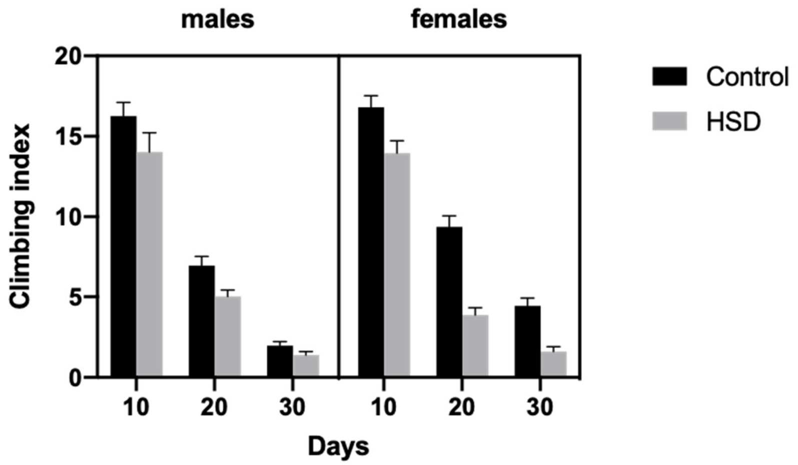

Figure S4. The climbing ability of w[1118] *Drosophila melanogaster* being reared on different diets and time periods. Bars show the mean climbing index of male and female flies being reared on a control and a high sugar diet (HSD) for 10, 20 and 30 days, respectively. Data have been subjected for multiple comparisons to a mixed-effects analysis. Data are presented as mean ± SEM from three biological replicates (n = 75).

Table S4. Results for mixed-effects analysis performed by GraphPad Prism

	Mixed-effects analysis<br>Tabular results				
1	Table Analyzed	Grouped_HSD_Climbing_all			
2					
3	**Mixed-effects model (REML)**	Matching by factor: Diet			
4	Assume sphericity?	Yes			
5	Alpha	0.05			
6					
7	**Fixed effects (type III)**	**P value**	**P value summary**	**Statistically significant (P < 0.05)?**	**F (DFn, DFd)**
8	Time	<0.0001	****	Yes	F (2, 288) = 430.9
9	Diet	0.0373	*	Yes	F (1, 268) = 4.381
10	Gender	<0.0001	****	Yes	F (1, 288) = 53.49
11	Time x Diet	0.4431	ns	No	F (2, 268) = 0.8165
12	Time x Gender	0.0721	ns	No	F (2, 288) = 2.654
13	Diet x Gender	0.0025	**	Yes	F (1, 268) = 9.342
14	Time x Diet x Gender	0.2334	ns	No	F (2, 268) = 1.463
15					
16	**Random effects**	**SD**	**Variance**		
17	Subject	0.7701	0.5931		
18	Residual	4.174	17.42		
19					
20	**Was the matching effective?**				
21	Chi-square, df	0.3003, 1			
22	P value	0.5837			
23	P value summary	ns			
24	Is there significant matching (P < 0.05)?	No			
25					
26	**Data summary**				
27	Number of columns	2 x 2			
28	Number of rows (Time)	3			
29	Number of subjects (Subject)	294			
30	Number of missing values	20			
